# On the contemporary history of learning disability identification procedures—a systematic literature review (1960–2000)

**DOI:** 10.3389/fpsyg.2026.1748297

**Published:** 2026-06-23

**Authors:** Michaela Vogt, Bhumika Bandodker, Till Neuhaus, María José Viteri Paredes, Federico Waitoller, Özge Erşan, Simonetta Polenghi, Anna Debè

**Affiliations:** 1 Faculty of Education, Bielefeld University, Bielefeld, Germany; 2 College of Education, University of Illinois, Chicago, IL, United States; 3 Department of Pedagogy, Università Cattolica del Sacro Cuore, Milan, Italy

**Keywords:** contemporary history, identification procedures, learning disability, systematic literature review, test

## Abstract

This systematic literature review critically synthesizes the academic literature on learning disability (LD) identification processes between 1960 and 2000, highlighting dominant conceptual frameworks and methodological tensions. The study addresses a critical gap in the literature by focusing on the historical context and development of LD diagnostic procedures, which have predominantly been characterized by conceptual ambiguity, varying diagnostic approaches, and the evolving understanding of LD. Utilizing a corpus of 82 peer-reviewed articles from predominantly English-speaking countries, the review employs a thematic synthesis approach to identify three central themes: (1) conceptual ambiguity concerning definitions and criteria of LD, (2) diverse approaches and challenges in diagnostic procedures, and (3) the focus on prediction and prevention methodologies. The findings underscore the absence of a unified LD definition and the influence of interdisciplinary perspectives, highlighting the necessity for further reflection as well as disciplinary discourse. The study calls for further research into historical and international comparisons of LD identification processes, suggesting that such efforts could enhance global consensus and improve diagnostic practices.

## 1 Introduction

Within the context of standardized mass schooling (Ramirez and Boli, 1987; Neuhaus et al., 2021), some learners deviated from institutional norms in more pronounced form and for longer periods of time (Brill, 2019; Pfahl and Traue, 2013). This is particularly true for the dimension of academic achievement (Bakker, 2021) and, in the course of the 19^th^ and early 20^th^ century, raised the question which learners can be considered “educatable” within the regular school system and which population of learners should be transferred into the special education track (cf. Brill, 2019; Hofmann, 2019; Neuhaus and Vogt, 2025). With the emergence of the IQ-test in 1905 (White and Hall, 1980), a technology has been created to draw a line between regular and deviant learners. However, due to inadequacies regarding identification solely on the basis of IQ testing (Reschly, 1981), along with the emerging awareness that “the weak learner” can be a product of multiple influences outside of innate cognitive ability (i.e. environmental factors, temporary disturbances, upbringing etc.), alternative constructs have been discussed in the 1950s and onwards. This discussion primarily took place at the nexus consisting of psychology, special education, pedagogy, and medicine (Debè et al., 2024).

One central concept, which emerged from these discussions, has been the concept of “learning disability” (LD). LD has first been defined by psychologist Samuel Kirk—considered by some the “father of special education” (Thomas Jr, 1996)—in 1962 as “a retardation, disorder, or delayed development in one or more of the processes of speech, language, reading, spelling, writing, or arithmetic resulting from a possible cerebral dysfunction and not from mental retardation, sensory deprivation, or cultural or instructional factors” (Kirk, 1962, p. 263). The concept quickly gained traction and “experienced unprecedented growth” exerting a long-lasting impact on the field of special education (Kavale and Forness, 2000, p. 239) as LD is “the most frequently identified class of disabilities among students in public schools” (Lyon et al., 2001, p. 259). Simultaneously, LD is also considered “the most problematic classification because of the vagaries and antagonisms surrounding definition” (Kavale and Forness, 2000, p. 239). Summarizing, it can be argued that the concept of learning disability seems to be an idea whose time has come as “the term ‘learning disabilities' caught on and swept the country—long before we had reached a really satisfactory definition of what it means” (Ames, 1977, p. 328).

In the light of these dynamics, this paper aims at mapping (Miake-Lye et al., 2016) and analyzing the research literature focusing on a selected facet of this topic, namely the identification processes in the field of learning disability^1^. In order to cater toward this goal, a systematic literature review (Nightingale, 2009; Xiao and Watson, 2019) will be conducted, with the period under investigation ranging from 1960 to 2000. Considering already existing literature reviews^2^ on LD identification processes, the state of research can be characterized as not overly pronounced as only few reviews have addressed the topic of assessment procedures and practices (see Anthony et al., 2024; Williams et al., 2016); however, these reviews have not been conducted systematically and largely ignore the historical dimension of identification processes. As to the knowledge of the authors, the only systematic literature review on identification procedures of learning disabilities has been presented by (Nehra et al. 2024) focusing on more recent studies being published from 2004 onwards. As such, unlike existing reviews that focus on assessment practices in isolation, the present study offers a historically grounded synthesis of research on LD identification procedures and can therefore not just be considered necessary but also timely as an emerging debate regarding the quality and reliability of assessment procedures can be observed in the international literature (see exemplarily Gasteiger-Klicpera et al., 2025; Rapp and Corral-Granados, 2024; Neuhaus and Vogt, 2022).

In order to cater toward this paper's aim, the larger goal—the mapping and analysis of existing research on learning disability identification procedures between 1960 and 2000—is compartmentalized into the following two research questions:

- RQ 1: How can the publications on learning disability identification processes be characterized regarding their medium of publication, their country of origin, as well as their disciplinary background?

- RQ 2: Which themes have been discussed in the literature on learning disability identification processes during the period under investigation?

In order to address these research questions, the article is structured as follows: Firstly, a brief overview on the applied methodology—including the approach toward the systematic literature review and the process of corpus construction (searched databases, search terms/ strings as well as inclusion/ exclusion criteria)—will be sketched out (section 2). Following this, the answers to the research questions that include a descriptive part—outlining the scope of research and further describing the constructed corpus—will be provided (section 3.1.), before the thematic analysis' findings are presented (section 3.2.). The article ends with a reflection upon this endeavor's limitations (section 4), a section of generated findings and conclusions (section 5) as well as recommendations for future research (section 6).

## 2 Methodology and corpus construction

In the following chapter, three individual aspects will be discussed. Firstly, a short introduction on systematic literature reviews will be provided in which not just the virtues of the method but also its usefulness for the research question at hand will be explored. Additionally, the necessity for this study will be further argued for, now also considering methodological aspects. Secondly, the different facets of the corpus construction (search for literature, inclusion/ exclusion of literature, and quality control) will be elaborated on before, thirdly, the approach toward the data analysis will be described.

### 2.1 The systematic literature review—methodological concerns

**Figure 1:**
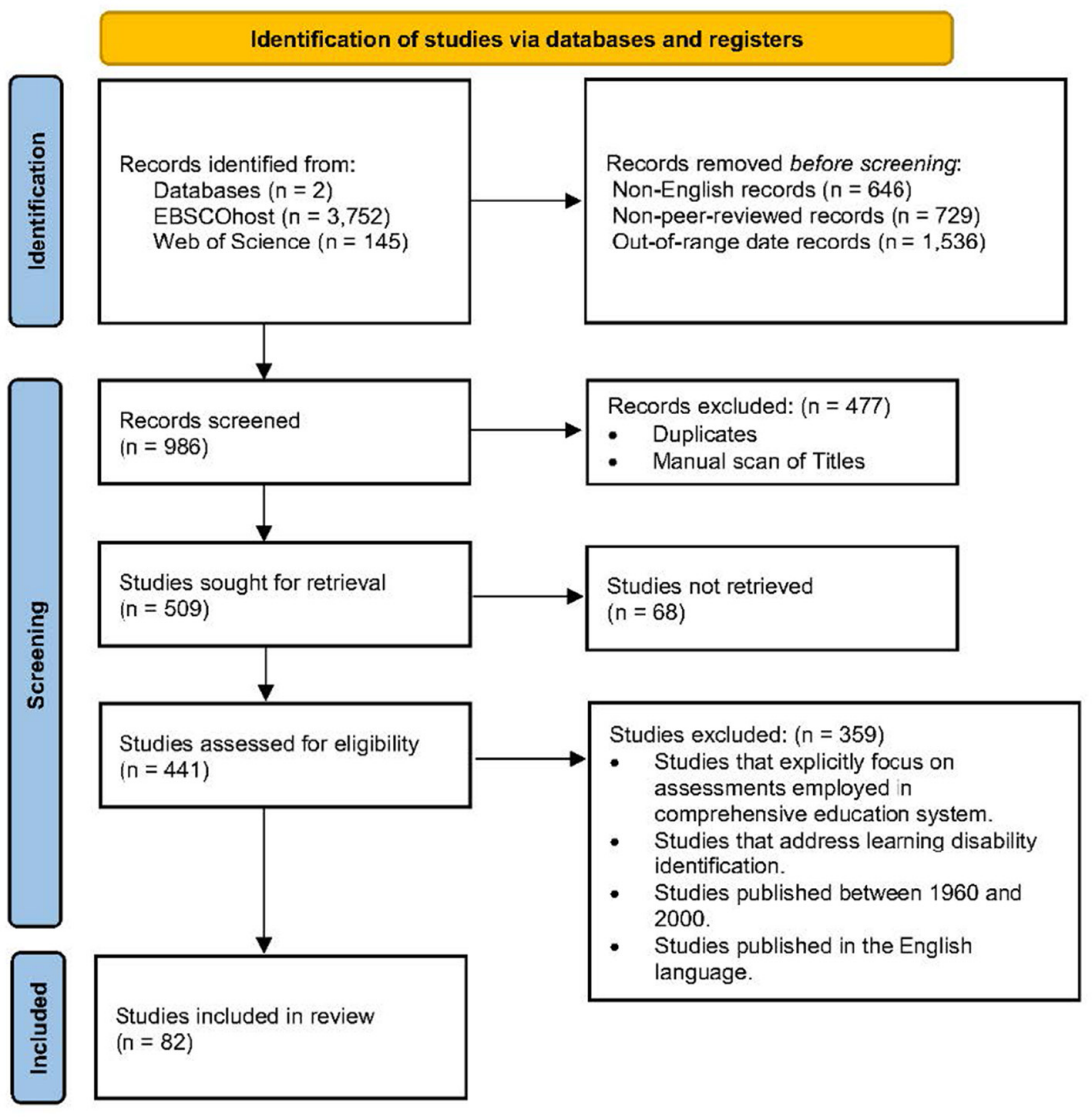
PRISMA flow diagram. PRISMA 2020 flow diagram for new systematic reviews which included searches of databases and registers only (Haddaway et al., 2022; Page et al., 2021).

Generally, the value of systematic literature reviews is primarily suspected in two domains: (1) evaluating interventions' effectiveness in order to inform future practices and approaches (Newman and Gough, 2020; Xiao and Watson, 2019) as well as (2) mapping existing research highlighting under-researched topics and perspectives (Farias-Gaytan et al., 2022; Pedersen et al., 2020; Soaita et al., 2020). The here presented study considers itself to be a mapping^3^ or cartography endeavor and thereby falling into the latter of the two categories “attempt[ing] to make sense of a body of existing literature through the aggregation, interpretation, explanation, or integration of existing research” (Xiao and Watson, 2019, p. 94). Systematic literature reviews of this kind have experienced heightened interest in recent times and have established themselves as common practice in educational science, especially in the subfields of special and inclusive education (see exemplarily Hernández-Torrano et al., 2022). As such, systematic literature reviews can facilitate rigorous explorations of existing research while also ensuring transparency and reproducibility (Newman and Gough, 2020). In order to cater toward these standards, (Page et al. 2021) suggest that a systematic literature review is built upon a high degree of description regarding methodological details, hence the Preferred Reporting Items for Systematic reviews and Meta-Analyses (PRISMA) framework and its corresponding flowchart (Haddaway et al., 2022) is used to report on the systematic inclusion and attrition of literature (see below, Figure 1).

The necessity for this systematic literature review (Nightingale, 2009; Xiao and Watson, 2019) can be justified by considering the conceptual and diagnostic ambiguity hinted at by Lyon (2001, p. 259) as “many disagree about the definition and classification of LD; the diagnostic criteria and assessment practices used in the identification process; the content, intensity, and duration of instructional practices employed”. This led some scholars to characterize LD as “a catch-all classification for a general class of learning problems” (Kavale et al., 2009, p. 52). Furthermore, the period under investigation from 1960 to 2000 can be considered a given as Samuel Kirk's attempts to define LD date back to the early 1960s. Regarding the ending of the period under investigation it can further be argued that after the year 2000 “[t]he focus shifted to the operational definitions […] necessary for the practical purpose of identification” (Kavale et al., 2009, 46). As such, the field of learning disability research could benefit from mapping the existing literature within the period under investigation and thereby become more aware of its dominant discourses, challenges, and understandings (Hernández-Torrano et al., 2022).

### 2.2 Corpus construction

#### 2.2.1 Search strategies

For this study, two databases have been searched, namely EBSCOhost^4^ and Web of Science. The search strings were developed with search terms that could be considered of high relevance for the period under investigation as well as the topic at hand. The individual parts of the search string have been isolated by an initial screening of preliminary literature, starting with (Kirk 1962) and ending with articles from the late 1990s. The employed terminology was further refined through exchanges between the authors. The goal at the review's initial stage was for the search to be expansive, only to be refined later through the screening and quality assessment processes. The following two search strings have been developed:

- String 1 (sen or special needs or special educational needs or special education) AND (assessment process or assessment procedure or diagnosis or identification)
- String 2: (assessment process or assessment procedure or diagnosis or identification) AND (learning disabilities or learning disorders or learning difficulties or learning disabled or feeble-minded or mentally retarded or subnormal or mental retardation).

Literature searches were carried out between November 2024 and March 2025, and yielded an initial total of 3,897 records from the databases.

#### 2.2.2 Criteria for inclusion/exclusion

After excluding articles which did not undergo a peer-review process, have not been authored in English, and have not been published within the period of investigation, a total of 986 articles remained for screening. This preliminary corpus has further been reduced to 441 records by excluding duplicates as well as text which were not accessible. At the next stage, the titles and abstracts of each retrieved text were screened for eligibility based on the following two, content related, inclusion/exclusion criteria:

- The article focuses on assessment procedures being employed in the comprehensive educational system
- The article discusses aspects from the realm of learning disability (or derivatives thereof^5^)

Articles had to fulfill all five criteria (1. peer-reviewed, 2. English, 3. published between 1960 and 2000, 4. focusing on LD, and 5. discussing assessment within the comprehensive schooling system) in order to be eligible for the final corpus. Applying all five criteria ensured that the final corpus was closely aligned to the scope of the research. Leaving out any one criterion would have broadened the corpus to include a wider range of disabilities (physical disability, specific learning disability etc.) manifesting both within and beyond school systems, thereby diluting the focus of the academic literature of interest. Furthermore, the methodological specifications for the articles were kept broad and thereby include qualitative, quantitative, and mixed methods studies. The screening stage reduced the pool to about 90 articles, which were then selected for full-text screening. Following quality checks and a final assessment of thematic appropriateness, 82 articles were retained and constituted the final corpus.

#### 2.2.3 Quality assurances

As a first measure of quality assurance, the screening of the articles was undertaken by two researchers who independently evaluated the appropriateness of the articles for the study based on the five inclusion/exclusion criteria (see section 2.2). Articles for which both researchers reached consensus were duly included. In cases of disagreement, the articles were brought to the larger research team for discussion, and suitability was determined collectively to ensure rigor in the screening process. Another layer of quality assurance was implemented by exclusively including journal articles stemming from peer-reviewed journals, owing to their recognized trustworthiness within academia and the thorough review processes they undergo (Nicholas et al., 2015). As a final measure, the methodological quality of 20 randomly picked articles from the final corpus has been assessed using a quality assurance checklist^6^. Three reviewers independently applied the modified checklist, which evaluates aspects such as clarity of research aims, appropriateness of methodology, data collection and analysis rigor, ethical considerations, and the clarity of reporting. Each of these items was marked (“Yes”, “No”, or “Can't tell”), and finally the studies were rated overall (e.g., high, medium, or low quality). All 20 articles were rated as either high or of medium quality, with none classified as low quality by the reviewers, so no further exclusions were required.

### 2.3 Data analysis

The analysis employs a two-fold approach to address the research questions individually—first through a predominantly quantitative synthesis, and subsequently through a qualitative and theme-oriented lens—thus constituting a mixed methods design. In the first stage of the analysis, the entire corpus has been analyzed using descriptive quantitative methodology and has been characterized with respect to several dimensions, such as the following: country of origin, journal names, publication date, and disciplinary orientation. This is in accordance with the guidelines for systematic literature reviews, stipulating that the “presentation of study results using tabulation and visual display is important for transparency and facilitates the identification of patterns in the data” (Page et al., 2021, p. 13). As such, by drawing from the repertoire of descriptive statistics as well as bibliometric analysis, the bibliographic data on the corpus of learning disability identification procedures has been organized in a manner in which the dataset can be analyzed, visualized and interpreted (cf. Zawacki-Richter et al., 2019). The results of this stage of analysis, addressing the first research question, are presented in section 3.1.

For the second research question, integrating diverse study designs constitutes a methodological challenge (Newman and Gough, 2020; Turnbull et al., 2023). Accordingly, it was necessary to adopt an integrative approach, such as thematic synthesis, in which “themes are extracted from the literature, clustered, and eventually synthesized into analytical themes” (Xiao and Watson, 2019, p. 101). These themes are then, in turn, used to answer the research question. The process of thematic synthesis in review studies has been summarized by (Tuckett 2005) as following:

The essence of analysis [...] involved the comparison of pieces of data judged to belong to a particular theme, in an effort to recognise the common feature of that theme. Incidents or events were coded (labelled) in terms of as many themes as were relevant and then these incidents or events within the theme were compared. The aim was to develop a set of logical themes and associated characteristics (exemplified by sub themes which together formed a ‘story'). Ongoing developmental analysis meant that the themes became guides for further analysis of data as the themes in turn were integrated through theorising. Such a process meant that early themes were often subsumed within this unification of ideas. (p.76)

As outlined by (Thomas and Harden 2008, p. 1), “[t]hematic synthesis has three stages: the coding of text ‘line-by-line'; the development of ‘descriptive themes'; and the generation of ‘analytical themes”'. For the specific case of this study, the coding has been conducted by three researchers^7^ using the MAXQDA software. Following the development of descriptive themes (cf. Thomas and Harden, 2008), iterative discussions were employed to reconcile conflicts, to validate themes, to ensure inter-coder reliability (O'Connor and Joffe, 2020, p. 2), as well as to synthesize the findings (Clarke and Braun, 2017). This process culminated in three analytical themes—each consisting of multiple descriptive themes—, which may help to (re-)construct the topography of the research landscape regarding learning disability identification processes.

**Figure A1:**
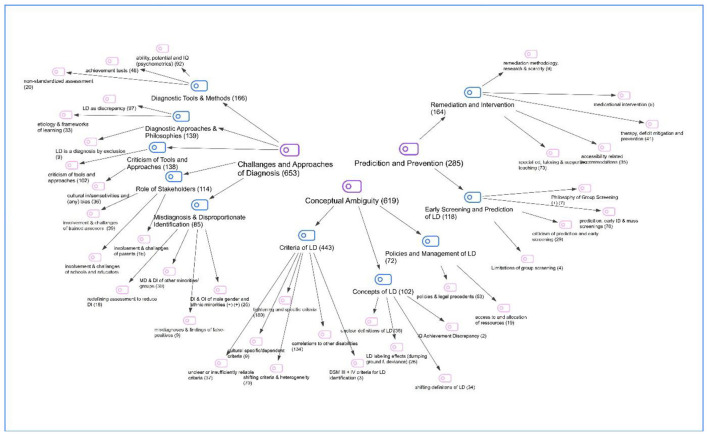
Thematic code tree. The figure illustrates the Hierarchical Code-Subcodes Model generated using MAXQDA.

Details of the coding framework are presented in Figure A1, illustrating the thematic code tree generated through MAXQDA. At the base of the tree are the line-by-line codes, which capture the ideas and recurring patterns identified in the primary texts. These initial codes were subsequently clustered into 10 descriptive themes that represent broader categories of meaning within the corpus data under review. The hierarchical structure of the code tree hence illustrates the progression from the codes to descriptive patterns and, ultimately, to analytical themes that conceptualize the state of scientific discourse on learning disability identification.

Each of these analytical themes will be sketched out in section 3.2, not just elaborating the descriptive themes which ultimately formed the analytical theme but also discussing their relatedness in order to “‘go beyond' the primary studies and generate new interpretive constructs, explanations or hypotheses” (Thomas and Harden, 2008, p. 1).

## 3 Findings

The findings are presented in line with the research questions outlined earlier and are organized in two main parts. First, by applying descriptive quantitative methodology, a corpus description and characterization will be provided. This description and characterization will address the first research question by outlining the scope of the research and describing the constructed corpus in terms of publication patterns, geographical origins, and disciplinary affiliations. Second, the thematic analysis of the corpus addresses the second research question by discussing the three analytical themes that emerged from the literature.

### 3.1 Quantitative characterization of the corpus

**Figure 2:**
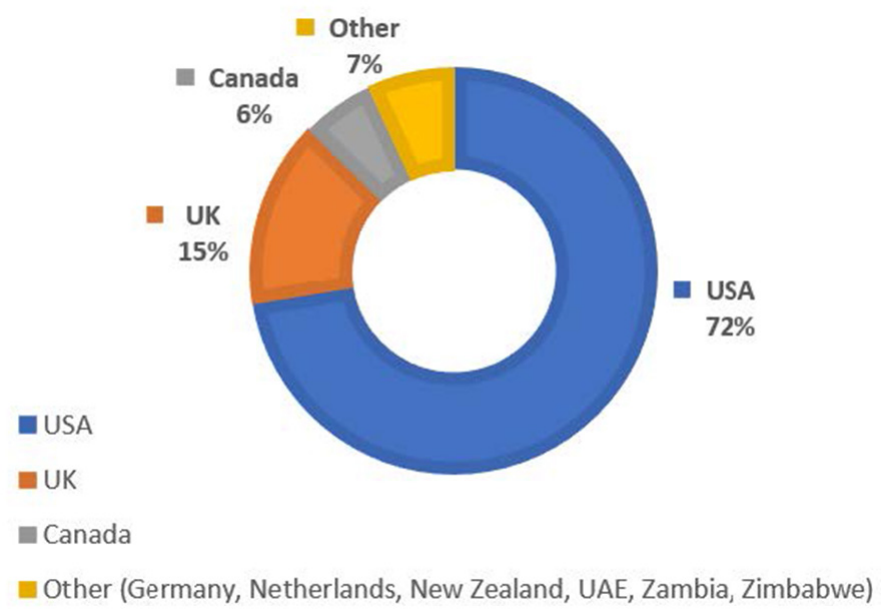
Distribution by country attribute.

**Figure 3:**
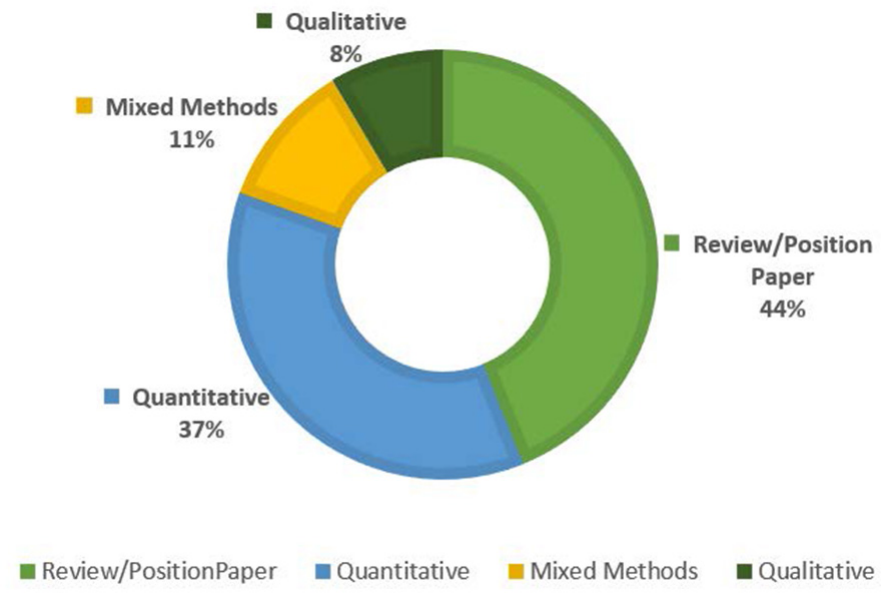
Distribution of research designs.

**Figure 4:**
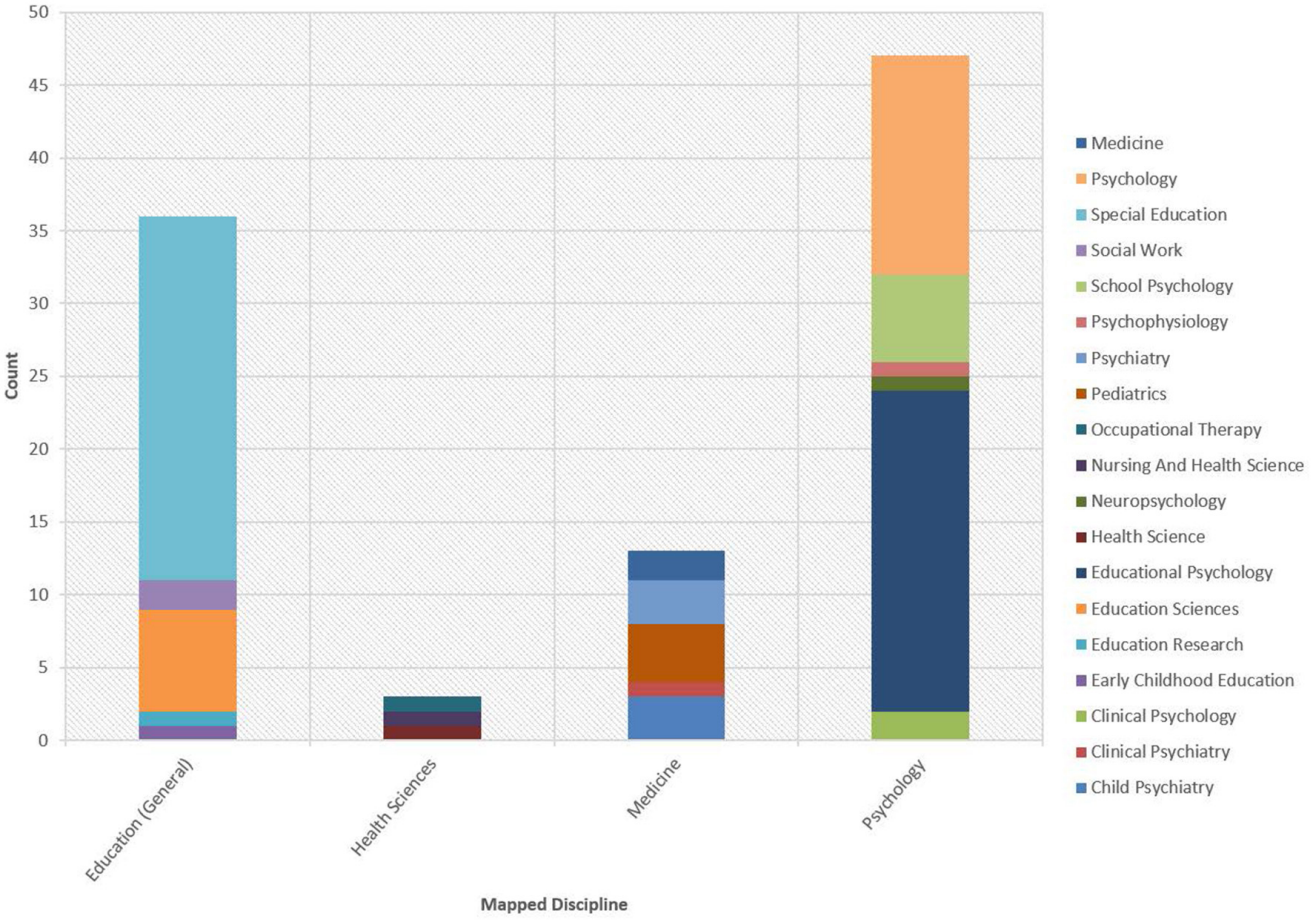
Distribution by disciplines. In this figure, interdisciplinary publications were added independently to each discipline, thereby producing mapping totals that exceed the final number of articles in the corpus.

The analyzed corpus consists of peer-reviewed journal articles published between 1960 and 2000. Figures 2–4 present the bibliometric dimensions concerning the country attribute, research designs and disciplinary orientation respectively. For the purposes of this analysis, the first descriptive dimension “country attribute” subsumes works that commonly denote the empirical context of the study characterized by the geographical focal point of the research interest. In cases in which the geographical focal point is not made explicit, the author's institutional affiliation has been taken as a proxy. The publications are predominantly composed of contributions^8^ from the USA (62), followed by the UK (13) and Canada (5), while countries such as New Zealand, the UAE, Germany, Zimbabwe, Zambia, and the Netherlands appear only once in the corpus. The dominance of USA-based publications suggests that LD identification has historically been shaped by Anglo-American epistemological frameworks indicating a strong concentration of foundational scholarship emerging from the Global North^9^.

Regarding the methodological dimension, the corpus can be divided into empirical studies and those of a more theoretical or conceptual nature. Among the empirical studies, 30 employ quantitative designs (37%), 9 utilize mixed-methods approaches (11%) and 7 adopt qualitative approaches (8%). The largest share of the total publications (44%) has been categorized as position papers or review articles, and fell within the scope of a theoretical or conceptual discussion on LD Identification. The high proportion of position and review papers could suggest that—during the period under investigation as well as in different scientific communities—there has been ongoing and controversial debate about the nature and conceptualization of LD.

By categorizing the corpus on the basis of disciplinary alignment, the discipline of each article was inferred from either the authors' academic background and/or institutional affiliation or—in cases when the background has not been mentioned and/or could not be inferred—the journal in which the article was published served as a proxy. Figure 4 illustrates that most papers originate from disciplines stemming from the psychological realm with a particularly strong presence of educational psychology and school psychology. This disciplinary dominance may have had implications for the conceptualization and operationalization of LD which diminished alternative disciplinary readings (for an historical account, see Stieger and Tröhler, 2023; Tröhler, 2013). The next largest contribution comes from the education field (e.g., special education, educational sciences and education research), followed by medical disciplines (e.g., pediatrics and psychiatry) and health sciences (e.g., occupational therapy and nursing). Several publications (*N* = 17) can be characterized as interdisciplinary spanning more than one field and reflecting the interdisciplinary nature of research on LD identification processes.

**Figure 5:**
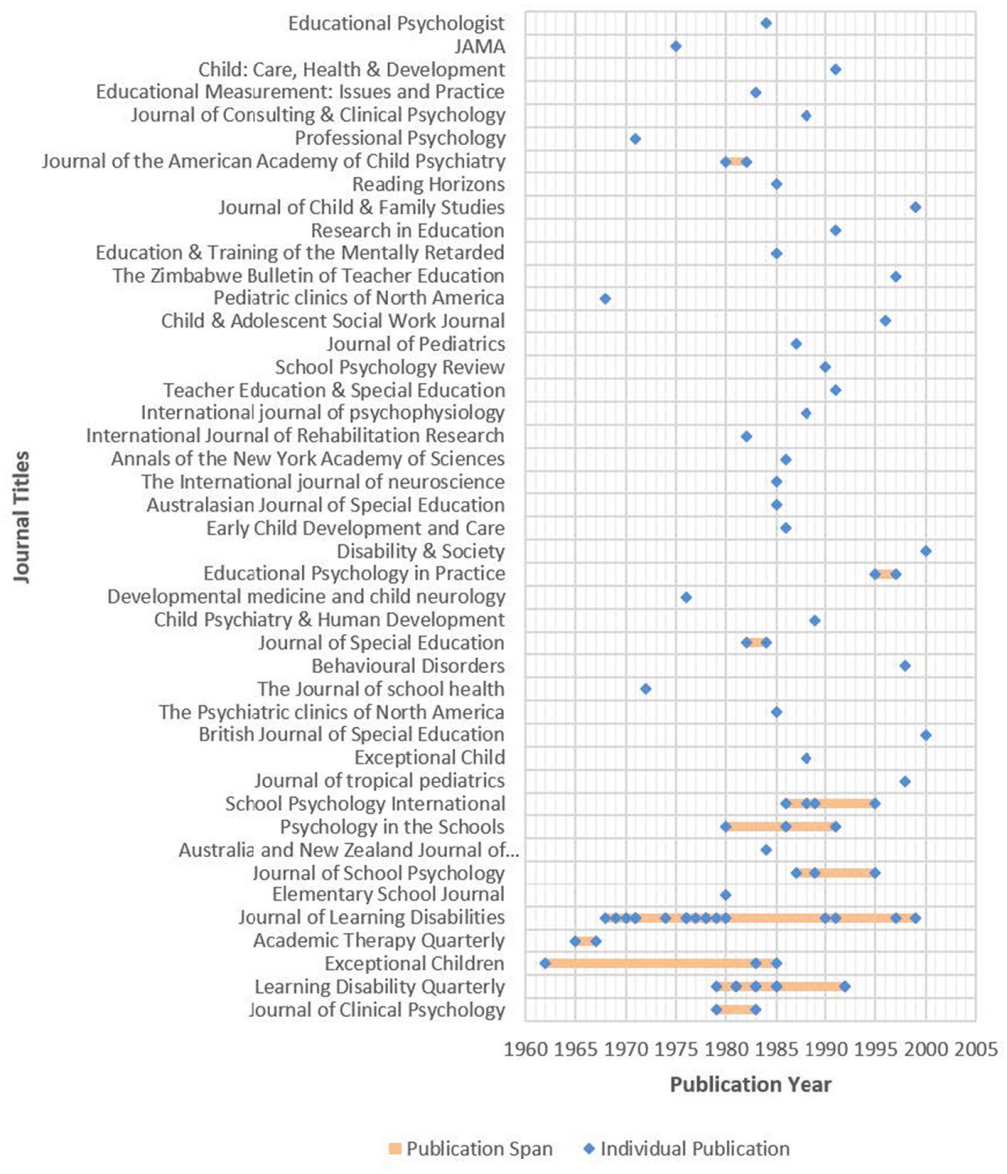
Distribution of publications by year and journals.

Moreover, as illustrated in Figure 5, the journals contributing the largest shares to the distribution are the *Journal of Learning Disabilities* (17 articles) and *Learning Disability Quarterly* (9 articles). Both are US based publication outlets and are dedicated to topics related to LD, mostly within the context of US states, policy, and service delivery. Other prominent journals featuring more than one article on learning disability identification procedures are represented by orange bars in the figure above, also illustrating the publication timeline or span of these articles. Regarding the publication years within the corpus, publication activity peaked in the 1980s, especially in the mid-1980s, suggesting a “boom” period regarding the output of scientific literature on LD identification procedures. This surge arguably reflects policy and practice shifts in the United States following the Individuals with Disabilities Education Act (IDEA) of 1975 (Reynolds, 1992) and, in the United Kingdom, the Education Act of 1981 (Pumfrey and Ward, 1991). Both special education-oriented policies acknowledged and introduced provisions for learning disabilities within their respective contexts in the late 1970s to early 1980s. These policy and practice shifts appear to have sparked heightened academic interest as well as discourse which resulted in an increased publication volume as observed in the findings.

### 3.2 Thematic description

As outlined in section 2.3, the corpus has been analyzed with the aim of generating descriptive themes which have been, in a second step, merged into analytical themes. In order to present and sketch out this study's findings, each analytical theme will be illustrated in terms of its general dynamic as well as the underlying descriptive themes. By presenting and discussing the analytical themes, it is attempted to ultimately answer the research question. The three analytical themes have been named as following: (1) Conceptual ambiguity, (2) approaches and challenges of diagnosis, and (3) prediction and prevention^10^. It is important to note that each of these analytical themes is—at different points and to diverging degrees—related to the other themes; connections have been drawn where applicable.

#### 3.2.1 Conceptual ambiguity

The first analytical theme which emerged from the analysis of the corpus has been entitled *conceptual ambiguity*. Studies being part of this theme discuss (1) *concepts of learning disability*, (2) *criteria of learning disability*, and (3) *policies and management of learning disability*.

The descriptive theme of *concepts of learning disability* (1) subsumes studies, position articles, and conceptual articles which try to advocate or reject—sometimes with reference to empirical positions and findings—a given understanding of learning disability. Prominent positions are the IQ-achievement discrepancy model (Merrell and Shinn, 1990; Reynolds, 1992; Rudel, 1980; Sabatino, 1971; Willson and Reynolds, 1984), the demarcation from alternative defects (i.e. mental retardation, cf. Aaron, 1979; Kirk and Bateman, 1962; Rudel, 1980; Palfrey, 1987), or definitions which primarily focus on educational non-achievement (cf. Shepard and Smith, 1983; Reynolds, 1985; Willson and Reynolds, 1984). One key pattern which can be identified in this part of the corpus are the constant attempts to establish a certain marker or definition, just to be falsified by other authors on the basis of empirical findings. As such, different kinds of correlations are presented, i.e. of learning disability and brain constitution/ injury (cf. Aaron, 1979; Rothstein, 1982; Rudel, 1980), retardation (Kinsbourne, 1986; Polloway, 1985; Swanson, 1984), non-verbal learning disability (cf. Palombo, 1996) or hyperactivity (cf. Aaron, 1979; Sulzbacher, 1975). The aspect of unspecific identification criteria has especially been discussed in the article authored by (Shepard and Smith 1983) in which low-achieving students have been compared to students being labeled as learning disabled. Surprisingly, the study could not show that the two groups do feature a clear line of demarcation. Observations of this sort have tempted other authors to characterize the term learning disability as a “dumping ground” (Beers and Beers, 1980, p. 69) or its criteria as “variant and nebulous” (Shepard and Smith, 1983, p. 1), which is employed when all other categories no longer seem valid. (Shepard and Smith 1983) also discuss the association of the term learning disability with “problem students” who:

[ought to be removed] from the regular classroom [...] Some of [the assessed children would show] minor behavior problems as their only abnormal characteristic. Some had complete files, but not a single indicator of LD or other learning or behavior problems. (p. 10)

Such dynamics led (Forness and Cantwell 1982) to the attempt to replace the, in their view too vague, pedagogical category of learning disability—one key finding is that learning disability is almost always considered a disability which presents itself in relation to an institution (mostly school) (cf. Reynolds, 1992)—with the more medical terminology of the DSM 3 manual, which “provides for the medical diagnosis of learning disability through the DSM-III-R categories of Specific Developmental Disorders (SDD) in content areas generally consistent with PL 94-142” (Reynolds, 1992, p. 3). Almost needless to say that this attempt did not result in widespread adoption of the suggestion, as “the medical diagnostic criteria for SDD are not acceptable to many public school agencies” [...] and “diagnosis based on these criteria [would usually] not entitle their child to services” (Reynolds, 1992, p. 3).

Very closely related to the already discussed *concepts of learning disability*, yet slightly differing in scope, is the descriptive theme *criteria of learning disability* (2). Analogously to the academic debates arguing for or against a certain understandings and definitions of learning disability, a similar tendency can be observed in the realm of criteria. While some studies focus primarily on educational markers (i.e. failing in school cf. Thomas, 1965, p. 82; Pumfrey and Ward, 1991, p. 63) other studies aim at further differentiating the group of learning-disabled children. Such attempts of differentiation involve the consideration of IQ—either as a defining factor of the group (cf. Clarizio and Phillips, 1989; Aaron, 1979; Reynolds, 1992; Polloway, 1985) or in opposition as the IQ-discrepancy model suggests (cf. Braden, 1987; Shepard, 1983; Reynolds, 1992; Rudel, 1980). Additionally, an array of studies discusses cut-off points regarding metrics in order to demarcate the population of learning-disabled children from regular learners but also from more severely affected subpopulations (i.e. mentally retarded children cf. Kinsbourne, 1986; Rudel, 1980; Polloway, 1985; Swanson, 1984). As such, different kinds of correlations are presented, e.g., of learning disability and brain constitution/ injury (cf. Aaron, 1979; Rothstein, 1982; Rudel, 1980), retardation (Kinsbourne, 1986; Kirk and Bateman, 1962; Polloway, 1985), non-verbal learning disability (cf. Palombo, 1996) or hyperactivity (cf. Aaron, 1979; Sulzbacher, 1975). Bringing these results together with the ones from the first descriptive theme (*concepts of learning disability*), it can be argued that similar dynamics are at play, namely a conglomerate of scholars trying to establish a given concept—and alongside it, criteria—of learning disability. Considering the analyzed corpus, no such concept-criteria coupling has established itself permanently.

Therefore, it could be argued that the collective search for a unifying definition as well as coherent and reliable criteria of learning disability has not produced the desired results. These dynamics have been summarized by (Kavale and Nye 1981) when they state that:

[...] LD research literature reflects a lack of consensus regarding standard identification criteria for designating an LD sample. Although major criteria traditionally found in LD definitions were present, the [reviewed] research literature was marked by diversity regarding the nature and prevalence of criteria used for LD identification. This diversity results in heterogeneous LD samples and a generic designation for a wide assortment of children. (p. 4)

Additionally, (Siegel 1999, p. 2) verbalizes general skepticism regarding the quality and reliability of testing mechanisms and procedures as the following quote indicates:

Much of the research in the field relies on standardized tests and identifies the learning disabled as those individuals who in some subjects perform significantly below average for their age cohort. There is an odor of tautology in that approach: How do we know that a low score or even a series of low scores implies an underlying lack of ability and not, for example, a lack of interest or motivation.

This conundrum surrounding concepts and criteria is further discussed and complicated in the third descriptive theme, *policies of learning disability* (3). Phrases and segments being assigned to this code mostly discuss newly emerging political definitions and guidelines. The relationship between policies of learning disability and the other two themes can be characterized as ambivalent. On the one hand, newly issued guidelines and political attempts to define learning disability change the population of students and shape the relevant criteria for being considered learning disabled. Some studies or, more precisely, sections thereof reflect on these changes and/or compare newly passed laws with their legal precedents (Desforges, 1995; Furlong and Yanagida, 1985; Kirk and Bateman, 1962; Reynolds, 1992). The writings of (Pumfrey and Ward 1991, p. 61/62) exemplarily illustrate the legal dimension and how it attempts to approximate the concept of learning disability as the following quote illustrates:

[U]nder the Education Act 1981 [in the UK], various category labels were replaced by the generic term ‘Special Educational Needs'. In turn, this led to two other important concepts. These are as follows in Section 1 of the Act: (a) A child has ‘special education needs' if he or she has a learning difficulty which calls for special educational provision to be made for him, (b) A child has a ‘learning difficulty' if he has a significantly greater difficulty in learning than the majority of children of his age, or he has a disability which either prevents or hinders him from making use of the educational facilities of a kind generally provided in schools and (c) ‘Special educational provision' means provision which is additional to, or otherwise different from, the educational provision made generally for children of his age.

On the other hand, changes in legislation are almost always under-complex as they will never cover all aspects of a complex concept and result, at least for some, in unintended consequences. Articles, segments, and codes analyzing and discussing shortcomings of political legislation as well as implications for certain student groups have also been assigned to this theme (cf. Furlong and Yanagida, 1985; Reynolds, 1992; Wood, 1991). Again, exemplarily, the article by (Reynolds 1992, p. 2) highlights such dynamics when he states that

[t]he oft-repeated, oft derisively cited, apparently poorly understood federal definition [U.S. Public Law 94-142, Individuals with Disabilities Education Act (IDEA), 1975], which states had to implement but were allowed to interpret, has been regarded generally as vague and too subjective. It is open to much interpretation and the courts have failed to provide clear guidance despite thousands of individual cases.

Additionally, it can also be stated that changes in legislation and policy (re-)vitalize research's interest to a certain extent. One such example may be the Individuals with Disability Education Act (IDEA) from 1975 which led to an increased output research-wise as illustrated in Figure 5. As such, policy documents can be considered cornerstones in an otherwise fuzzy research environment which, depending on their scope and perceived importance, serve as gravitational centers within discourses.

Summarizing, it can be stated that the theme of conceptual ambiguity vividly illustrates different disciplines' struggles to define learning disability and to agree on widely accepted thresholds and identification markers. Given the epistemic conundrum as well as the unspecified disciplinary affiliation of learning disability, the policy level also attempts to contribute and define learning disability from an administrative standpoint. These attempts not just result in a plethora of critical scholarship challenging administrative definitions but also enlivened academic interest in the issue resulting in higher output in terms of academic articles.

#### 3.2.2 Approaches and challenges of diagnostic procedures

The second analytical theme, entitled *approaches and challenges of diagnosis procedures*, is closely related to the findings in the conceptual ambiguity section, yet it focuses to a larger extent on the actual process of identification and less about the underlying empirical and theoretical assumptions. Overall, this analytical theme can be considered the largest in terms of assigned articles and segments (see Appendix). As the title of the theme suggests, it consists of two descriptive themes: Firstly, *approaches toward diagnostics and diagnosis* as well as, secondly, *critical perspectives on tests and testing*.

Regarding the descriptive theme of *approaches toward diagnostics and diagnosis*, the following three subthemes emerged: (1) diagnostic approaches and philosophies, (2) role of stakeholders as well as (3) tools and tests.

Under the first subtheme, articles have been subsumed which discuss the aims, functions, and rationale of learning disability identification processes. The question regarding the philosophical orientation of the processes could be described along a spectrum, consisting of—at the one end of the spectrum—pedagogical approaches toward identification and, on the other end of the spectrum, rather quantitatively-minded medical and psychological approaches. This spectrum and variations in approaches is exemplarily summarized by (Bateman 1967, p. 8) when she states that:

Earlier it was said that all three approaches—etiological, diagnostic-remedial, and task-analysis—can be utilized by one diagnostician. At this point it might be urged that all three should be used, as each has a potential contribution to make to our knowledge about learning problems, even though they might not contribute equally to educational planning per se. [...] All have a place, all have similarities, and all have a unique emphasis. The accomplished diagnostician is able to utilize the contributions and avoid the pitfalls of all.

The latter end of the spectrum, the quantitatively-minded medical and psychology side, argues that with more precise predictors and extensive testing regimes individual children could be analyzed and understood more thoroughly. Also, proponents of this perspective hope that with more precise numbers, the question regarding the etiology of learning disability could be answered (cf. Aaron, 1979; Alley, 1979; Braden, 1987). On the other side of the spectrum, namely the pedagogical perspectives on identification processes, arguments and approaches can be found which primarily focus on pedagogical work and the contributions diagnostic procedures could provide there. Proponents of this kind of thinking see less value in more precise procedures but rather advocate for diagnostic processes which feature pedagogical implications, such as the identification of certain weaknesses which could then be addressed through specific pedagogical interventions (cf. Desforges, 1995; Kirk and Bateman, 1962; Reynolds, 1992; Schaer and Crump, 1976). Also, the here described perspective of pedagogical approaches toward diagnosis and identification puts more emphasis on formative forms of assessment instead of a strict categorization—a continuation of the argument outlined prior (cf. Owen et al., 1976; Rhodes, 1985; Thomas, 1965; Stringer et al., 1997).

Apart from these two general approaches toward identification processes in the field of learning disability, also a third position can be distilled from the corpus; however, this position is—regarding its breadth and scope—less pronounced than the two earlier mentioned positions. This third position questions the usefulness of the identification process all together. Proponents of this position cite shortcomings and structural biases within these identification processes and—oftentimes arguing from a power-focused theoretical basis—try to delegitimize the endeavor of identifying learning disabled children. The reasoning of (Peresuh 1997, p. 53) can be read as one exemplary take of such a perspective:

One of the most serious objections to the use of I.Q. tests in developing countries is that few of them have been properly adapted and standardized on appropriate populations. It is highly misleading to interpret the score on such tests with reference to norms derived from a culturally alien standardization sample.

The second subtheme, *role of stakeholders*, discusses the involvement and role of different actors in identification processes. Generally speaking, three different kinds of people have been discussed under this code. Firstly, the role and involvement of the assessor as he or she not just requires certain skills and should have undergone specific trainings (cf. Siegel, 1999; Kirk and Bateman, 1962; LaPolla et al., 1982) but can also—due to unpreparedness, a lack of skill, or limited knowledge—influence the identification process in adverse fashion (cf. Palombo, 1996; Shepard, 1983). Secondly, the role of parents is discussed in this subtheme as parents can—through the provision of useful information (cf. Palfrey, 1987; Peresuh, 1997; Sulzbacher, 1975)—help in the course of the identification process or, in less fortunate circumstances, provide insufficient information. For the latter case, certain rationales—fear of labeling, criticism, or stigmatization (Polloway, 1985)—have been discussed. Lastly, articles in the corpus also discussed the role and involvement of schools and educators. This subtheme features research on administrative processes as well as potential improvements thereof (cf. Desforges, 1995; Satz and Fletcher, 1988; Shepard, 1983; Zavala and Mims, 1983). Also, the role of educators—as they usually initiate the identification process by writing referrals and transmitting knowledge about the child (cf. Rhodes, 1985; Schaer and Crump, 1976; Palfrey, 1987; Thomas, 1965)—has been discussed extensively in the research literature.

The third descriptive theme within the analytical theme of *approaches and challenges of diagnosis* has been coined *tools and tests*. Analogously to the theme of *criteria of learning disability*, different studies focus on or employ different tests. By establishing this code, the spectrum of employed tests and tools can be approximated. The analyzed studies mentioned the following kinds of tests: IQ-tests (i.e. WISC, Stanford-Binet, Woodcock-Johnson), achievement and attainment tests (i.e. Wide Range Achievement Test, Peabody Individual Achievement Test), (psycho-)linguistic tests (i.e. Illinois Test of Psycholinguistic Abilities), as well as a variety of unstructured and unstandardized testing methods (i.e. interviews, observation etc.). As stated earlier, the majority of studies employed more than one testing approach and/or consulted aggravated metrics. Also, criticism and shortcomings of tests have been discussed under this theme. Resonating criticism have been cultural biases and insensitivities regarding different minority groups (cf. Argulewicz and Sanchez, 1983; Braden, 1987; Desforges, 1995). This collection of testing batteries further illustrates the conceptual differences and illuminates which specific tools have been employed by different professions and professionals when attempting to identify learning disabled children. As such, theoretical and conceptual differences translate into different approaches and tools.

After having discussed approaches, tools, and stakeholders, the second descriptive theme of this analytical theme is focusing primarily on the *critical perspectives on tests and testing*. As already hinted at in the tools and tests section, identification procedures as well as the employed tests have been criticized on multiple grounds. These criticisms can be subsumed under two subthemes, namely (1) misdiagnosis and disproportionate identification and (2) criticism of tools and approaches. Disproportionate identification describes the numerical overrepresentation of certain marginalized subgroups. Examples of such groups are ethnic minorities (i.e., in the context of the USA, African American or the Latinx community, cf. Argulewicz and Sanchez, 1983; Braden, 1987; Zavala and Mims, 1983) or disadvantaged groups (e.g., in terms of socio-economic status, cf. Argulewicz and Sanchez, 1983; Pumfrey and Ward, 1991; Shepard and Smith, 1983). Such overidentification dynamics led scholars to the conclusion that the identification process or employed testing batteries are inherently racist, classists, or otherwise discriminating (cf. Braden, 1987; Peresuh, 1997). On the basis of such observations, some scholars advocate for the abolishment of identification processes or question their general validity and usefulness (cf. Sabatino and Miller, 1980; Peresuh, 1997; Shepard, 1983).

Additionally, the descriptive theme of *critical perspectives on tests and testing* also encompasses the subtheme of *criticism of tools and approaches*. This theme subsumes incidents and/or classes of people who have systematically been incorrectly diagnosed. The dynamic being referenced here oftentimes cites heuristics and biases in which individual facets, traits, or test scores outshine others and assessors are tempted into false diagnosis (cf. Palfrey, 1987; Shepard, 1983; Siegel, 1999); as such, the here described dynamics can be considered criticism of certain diagnostic tools and approaches. Summarizing, these subthemes can be considered severe criticism which highlight the weakness of identification procedures and question their epistemic authority (cf. Argulewicz and Sanchez, 1983; Sabatino and Miller, 1980; Peresuh, 1997; Shepard, 1983).

Summarizing, it can be stated that the here presented analytical theme—*approaches and challenges of diagnosis procedures*—can be read as a continuation of the first theme (*conceptual ambiguity*) as the publications within the corpus struggle to agree upon the purpose and procedure regarding the identification processes. This struggle manifests itself not just in different purposes—ranging from medical diagnosis to pedagogically-minded assessment—but also in a diverging valuation of stakeholders as well as testing batteries being suggested. The here described debate is further enlivened by findings which suggest that the identification processes deliver questionable results, such as overrepresentation of marginalized communities and/or misdiagnosis of specific groups of people.

#### 3.2.3 Prediction and prevention

The analytical theme of *prediction and prevention* is arguably the one theme which separates itself the most from the two aforementioned. Generally speaking, the theme consists of two descriptive categories, namely (1) *remediation and intervention* as well as (2) *early screening and prediction*.

The category of *remediation and intervention* (1) subsumes studies and articles which primarily focus on different kinds of interventions aiming at the improvement of specific skills and functions of learning-disabled children. These interventions encompass attempts to improve school-related skills, such as reading, writing, arithmetic (cf. Aaron, 1979; Reynolds, 1992; Schiller and Deignan, 1969), first and second language proficiency (cf. Desforges, 1995; Zavala and Mims, 1983), problem-solving and information processing (cf. Rudel, 1980; Stringer et al., 1997; Swanson, 1984), executive function (cf. Mangina and Beuzeron-Mangina, 1988; Palombo, 1996; Swanson, 1984), or motor skills (cf. Schiller and Deignan, 1969; Stephenson et al., 1991). Additionally, an array of studies also emphasizes the necessity to foster skills outside of the standard school-related domains. Studies of this type focus on the one hand on intervening on aspects underlying successful learning—examples of that would be emotional regulation (cf. Sulzbacher, 1975) or the improvement of meta-cognitive strategies (cf. Aaron, 1979; Kirk and Bateman, 1962; Swanson, 1984)—but on the other hand also covered aspects which could be characterized as often occurring co-morbidities of children diagnosed as learning disabled. The latter kind of studies discuss efficiencies of different interventions in fields such as behavioral issues (cf. Kirk and Bateman, 1962; Polloway, 1985; Schiller and Deignan, 1969) or the remediation of social circumstances (e.g., poor upbringing, lack of stimuli, or experiences of deprivation).

While the descriptive theme of *remediation and intervention* encompasses a plethora of approaches which exhibit some stand-alone qualities within the corpus, the second aspect of the analytical theme—the descriptive theme of *early screening and prediction* (2)—mirrors, at least to some extent, selected facets of the other two analytical themes (see 3.2.1. and 3.2.2.). Generally speaking, the category of early screening subsumes research which primarily focuses on the necessity and role of early identification of (potentially) learning disabled children. The efforts to identify critical indicators start before the child's entry into the comprehensive schooling system, ranging from kindergarten (cf. Palfrey, 1987; Rhodes, 1985; Thomas, 1965) to early childhood and infancy (Shapiro et al., 1984). Furthermore, the validity and role of group screening and its predictive capabilities are justified and critiqued by a few (Buktenica, 1971; Gipps and Gross, 1986). (Buktenica 1971, p. 4) discusses the issue of early screening and prevention as following:

And, finally, unless attempts at early identification and intervention use group screening and group intervention procedures, it seems unlikely that any semblance of prevention will be effected.

Studies being assigned to this descriptive category are oftentimes designed as follow-up or longitudinal studies (cf. LaPolla et al., 1982; Satz and Fletcher, 1988; Schaer and Crump, 1976) in which early behaviors, test scores (cf. Kinsbourne, 1986; LaPolla et al., 1982; Schaer and Crump, 1976), and further characteristics are then later connected to a diagnosed learning disability. Although promising the possibility of early intervention, early screening methods also present significant challenges. The biggest challenge as perceived through the analysis of the corpus seems to be the methodological validity and reliability (cf. Palfrey, 1987; Satz and Fletcher, 1988; Schaer and Crump, 1976). In that regard (Satz and Fletcher 1988, p. 828/829) come to the following conclusion in their study:

We are concerned over the increased laxity of some journals to publish reports on early screening, often lending credence to approaches, tests, or conclusions that are not based on sound methodologies. [...] Consequently, the gross errors in computation and the tendency to uncritically accept the results of incomplete validation studies have impeded progress in early detection and intervention. [...] The risks of inadequate screening, including the iatrogenic effects of mislabeling a low-risk child or the inability to intervene with an undetected high-risk child, are high. Consequently, children suffer when the methodology is insufficient.

In accordance with the findings subsumed in the other two analytical themes, a decentralized discourse can be observed in which different scholars and scientists argue for certain predictors or combinations of predictors to foresee a learning disability, just to be corrected by other scientists in the field. However, no single metric and/or set of combined metrics has had the epistemic authority to establish itself as the standard approach for early screening in the field of learning disability. However, studies within this theme highlight the necessity of early identification, especially to prevent children from developing adverse effects in their development and learning. As such, this category can be characterized as ambivalent as on the one hand the imperative of identifying and supporting potentially learning-disabled children as early as possible is issued paramount, yet, on the other hand, no consensus regarding the actual predictors could be identified.

While, at first sight, differing from the first two analytical themes, the theme of *prediction and prevention* still shares a tremendous amount of similarity with the already discussed themes as a plethora of preventive and supportive measures have been suggested. These measures loosely correspond to the disciplinary struggles outlined earlier and encompass psychological, medical, as well as pedagogically-minded approaches. Furthermore, the prediction of learning disability seems to feature similar insufficiencies as the diagnostic process in general as no combination of metrics or factors prevailed.

## 4 Limitations

The here presented study features, due to its applied search parameters, a couple of limitations which will be made transparent in the following section. Generally speaking, the major limitation of this study can be ascribed to decisions regarding the corpus construction. As outlined in the section “Corpus Construction” (see section 2.2.), certain inclusion and exclusion criteria have been applied in order to determine whether an article will be added to the final corpus or not. These criteria structure the process of corpus construction; however, they can also contribute to or cause certain blind spots within the corpus. Here, the authors would like to highlight two potential blind spots within the corpus and would emphasize the necessity for further research in these areas:

- Due to the fact that the articles had to be published in the English language in order to be eligible for the study, the corpus—and with it, the research contexts—are overwhelmingly stemming from the anglophone countries (particularly USA, Canada, Great Britain/ UK/ England, and Australia, see section 3 for an elaborate distribution of geographical research contexts). As such, approaches, knowledge cultures, and ideas from these geographies are overrepresented in the study. Simultaneously, knowledge, practices, and approaches from outside these geographies are underrepresented which, in turn, may contribute to a Western bias^11^.
- In order to be eligible for the inclusion into the corpus, a study had to be published in a peer-reviewed journal. This may have had two major implications. Firstly, this inclusion/exclusion criterion favors disciplines whose disciplinary cultures align with peer-review (cf. Baldwin, 2017). As such, it could be argued that certain disciplines are overrepresented as their mode of knowledge generation and dissemination aligned better with peer-review whereas other disciplines may not have been considered to the same extent as they favor different ways of publication (i.e. monographs, edited collection, gray literature etc.). Secondly, a focus on peer-reviewed studies is also neglecting the fact that publication habits have changed tremendously from the 1960s to the year 2000. As such, early adopters of current practices (i.e. a strong focus on peer-reviewed journals) may be overrepresented in the sample.

Apart from the here outlined limitations, the authors would also like to highlight a potential for a secondary analysis of the here presented corpus. As this paper's aim was to map out the field of learning disability identification processes, a second dynamic has been neglected: the prevalence of themes over time. While this can barely be conducted in a systemic literature review, it could be of interest—e.g., by applying a more discourse-sensitive method—to trace the developments over time and to illustrate the booms and busts of certain perspectives.

## 5 Conclusion

This paper attempted to map and analyze the existing research on learning disability identification processes being published between 1960 and 2000. This endeavor has been compartmentalized into two research questions:

- RQ 1: How can the publications on learning disability identification processes be characterized regarding their medium of publication, their country of origin, as well as their disciplinary background?

- RQ 2: Which themes have been discussed in the literature on learning disability identification processes during the period under investigation?

Regarding research question 1, the following findings can be reported:

- Research on learning disability identification processes has primarily originated from the anglophone countries (USA, UK, Canada) which not just constitutes an epistemic asymmetry but also caters to the fact that what is being understood as learning disability is heavily shaped by the discourses of the aforementioned countries. The prevalence of anglophone studies is also supported by the analysis of the medium of publications as large, anglophone journals have been the preferred place of publication; however, a disciplinary spectrum—ranging from highly specialized journals on LD to psychological as well as medical journals—could also be identified.
- Regarding the disciplinary background of the studies and authors, it can be stated that the different branches of psychological research constitute the largest share of research, followed by educational sciences. Medical research on learning disability also contributed to the discourse; however, in quantitative terms it constituted the smallest portion of the three mentioned disciplines.
- Additionally, the study designs have also been quantitatively measured. It could be shown that the largest share (44%) of analyzed papers consists of position/review paper; 37% of the corpus employ a quantitative design, 11% use a mixed-methods approach, and only 8% utilize qualitative research. As such, it can be concluded that, despite the dominance of quantitatively-minded psychological research in the field, the field apparently struggled to agree on unified definitions and criteria which may account for the expansive share of position and review papers.

The second part of the systematic literature review consisted of a thematic analysis in order to identify and sketch out themes within the literature on learning disability identification procedures. The thematic analysis isolated three analytical themes, namely (1) *conceptual ambiguity*, (2) *approaches and challenges of diagnosis*, and (3) *prediction and prevention*. Within these analytical themes, different dynamics as well as sub-themes could be identified which support the conclusion that—during the period under investigation—an on-going debate regarding the nature, etiology, measurement, and identification of learning disability took place. Ultimately, the analysis suggests that this debate did not result in a finite answer but rather different schools of thoughts, closely related to the disciplines involved (here: medical-psychological science and education science) established themselves. The here described debate permeates different facets of the analyzed corpus and highlights that the underlying, yet highly ambiguous and vague, concept (LD) resulted in debates within different sub-groups. As such, a variety of test formats, challenges, stakeholder perspectives, and approaches (including corresponding “testing philosophies”) could be identified. Apart from the sketched-out approaches and philosophies, an additional dimension could be isolated, namely the legal-administrative domain. It could be shown that within these highly sophisticated academic debates and processes of knowledge generation, legal changes attempted to create clarity regarding the nature and assessment of learning disability. While these attempts arguably resulted in heightened interest in the topic and contributed to higher publication outputs, the definitions and identification procedures provided by lawmakers remain under-complex and have sparked controversy within the academic domain.

Before outlining recommendations for future research, this paper will attempt to provide a theoretical re-perspectivize of the generated results. This will be done by employing the concept of the boundary object. From a theoretical lens, it can be argued that the concept of learning disability—with all its disciplinary re-interpretations, diverging national readings, and different local implications (Katchergin, 2014)—qualifies as a “boundary object” (Star, 1989) as it constitutes an artifact which serves a bridging function between professions, different research communities, and administrations. As such, learning disability enables a “process through which actors from different social worlds—called upon to cooperate—manage to coordinate with each other in spite of their differing points of view” (Trompette and Vinck, 2009, p. 5; see also Akkerman and Bakker, 2011, p. 134). This notion can be supported empirically by looking at the publication infrastructure of the corpus. It could be shown that the discourse of learning disability identification procedures involves many different disciplines and subdisciplines; however, a large share of the articles has been published in journals exclusively focusing on learning disability. These journals can be seen as the attempt to bridge disciplinary boundaries, traditions, and approaches by focusing on the phenomenon or boundary object.

And while these “[o]bjects or ideas may exist within boundary spaces as tools or common points of reference for interactions” (Crawford, 2024, p. 45), boundary objects are understood differently by each of the involved groups and actors; therefore, meanings need to be constantly negotiated (Star, 2010). This observation regarding boundary objects can also be identified within the corpus as extensive parts of the analysis highlight the struggles to establish a shared understanding of the phenomenon as well as consequences derived from that understanding, such as diagnostic procedures or interventions. This being said, it can be argued that learning disability could and arguably should be considered a boundary object.

## 6 Recommendations for future research

Based on the experiences as well as outcomes of this study, the authors would like to highlight the following conceptual, methodological, and content-related aspects as recommendations for fruitful future research:

- Firstly, it seems to be noteworthy that almost all analyzed studies operate under a national paradigm. Arguably, one result thereof is the fact that diagnostic categories and identification processes are only discussed, negotiated, and criticized from a national point of view and barely compared to or reflected against the backdrop of other nations' approaches. As such, future research in the field of learning disability identification procedures could and should adopt a more comparative framework. Such a framework could not just enable more thoroughly conceptual developments but could also contribute to a decolonization of knowledge in the field as—the at this point dominant—anglophone discourse could be enriched by alternative perspectives and knowledge (cf. Young, 2014).
- Secondly, from a methodological standpoint, the authors would like to highlight the opportunities for an expanded systematic literature review which, apart from journal articles, should also involve archival materials, gray literatures, policy recommendations, as well as individual chapters. Such an approach could cater toward a more holistic and less disciplinary biased understanding of learning disability identification processes while also allowing for the incorporation of a wider array of historical source material.
- Thirdly and lastly, it can also be observed that current studies almost always operate in the now-time thereby neglecting historical patterns and developments. This lack of historically-situated studies should be addressed in the future as concepts and diagnostic categories are not just demanding ideas but also shape current understandings and policies. Scholarship suggests that educational reforms will fail if they neglect the function and historical development of a given aspect (Labaree, 2021; Mehta and Datnow, 2020; Quesel, 2012). As such, a deeper investigation into the historical dynamics of learning disability identification processes may appear timely, not just for epistemic purposes but also to enable effective and historically-informed policy recommendations.

## Data Availability

The original contributions presented in the study are included in the article/supplementary material, further inquiries can be directed to the corresponding author.

## Footnotes

1 This focus has been selected as the actual identification process has a different degree of urgency due to the fact that real-life decision (including the potential consequences) has to be made. As such, we assume that the process as well as the research discussing these processes can be considered the application-oriented outcomes of the otherwise conceptual debates regarding the definition of learning disability.

2 It should also be mentioned that additional literatures on learning disability and its identification exist, such as intellectual histories (cf. Danforth, 2009), historical reconstructions and reflections (Jarrett and Tilley, 2022), or historically-oriented case studies (cf. Neuhaus and Vogt, 2026). All of these genres can be considered instructive for this study, yet they differ in their methodological approach (here: systematic literature review) as well as their unit of analysis (here: academic outputs/ research articles).

3 Technically speaking, a systematic literature review is not a set method but rather an umbrella term to describe a plethora of diverging review formats—Xiao and Watson (2019, p. 94) have therefore developed a “typology of literature reviews”. Within that ecosystem of literature reviews, this study considers itself to be a thematic synthesis (Thomas and Harden, 2008). Xiao and Watson (2019, p. 101) describe reviews from the family of thematic synthesis as following: “The data extraction and synthesis process for thematic synthesis utilizes thematic analysis; themes are extracted from the literature, clustered, and eventually synthesized into analytical themes. These analytical themes, similar in their construction to third order constructs, are then used to answer the research question.” The exact nature of this review as well as the specific steps it involves will further be clarified in section 2.3. (data analysis).

4 EBSCOhost can be considered a meta-database as it retrieves entries—in the field of education—of more than 20 other databases.

5 The search includes articles focusing on general learning disorders and “weak learning/learners”; however, highly specialized disorders – usually being subsumed under the label of specific learning disorders –, such as dyslexia, dysgraphia, or the alike, have not been included as they differ (i.e. regarding identification processes, prevalence, or intervention) qualitatively from generalized learning disability (cf. Peters and Ansari, 2019; McDowell, 2018; Vidyadharan and Tharayil, 2019). While controversial debates regarding learning disability and their demarcation from specific learning disabilities took place (cf. Kavale et al., 2009), it can still be stated that learning disability operates differently as specific learning disabilities define themselves in relation to “an important skill domain [that] is found to be somewhat dissociated from intelligence” (Stanovich, 1993, p. 502) while learning disability operate on a more general level.

6 A modified version of the CASP (Critical Appraisal Skills Programme) Checklist has been employed.

7 All three researchers come from the nexus of special education research and history of education.

8 Since certain articles are associated with multiple countries, as for instance the comparative studies by (Frederickson and Reason 1995), (Peresuh 1997), (Pumfrey and Ward 1991), and (Ysseldyke and Meijer 1986), the aggregate of the national counts reported exceeds the corpus's final total of 82 articles.

9 In part the over representation of US based publications has also been result of the search strategy of the SLR. This issue will be critically discussed in this article's limitations (see also Nehra et al., 2024).

10 In order to also depict the quantitative dimension of these themes, a numeric distribution of the descriptive and analytical themes has been provided (see Appendix Figure A1).

11 Despite the inclusion criteria, the dominance of Anglo-American scholarship in the corpus is consistent with well-established patterns of epistemic inequality in academic publishing (cf. Young 2014). Consequently, the prominence of Anglo-American epistemic frameworks in the findings was an expected outcome, reflecting the unequal structures through which academic knowledge has historically been produced, legitimized, and disseminated (cf. Tröhler, 2023).
